# Incisor and Soft Tissue Characteristics of Adult Bimaxillary Protrusion Patients among Different Skeletal Anteroposterior Classifications

**DOI:** 10.3390/diagnostics14101031

**Published:** 2024-05-16

**Authors:** Thitirat Siangloy, Chairat Charoemratrote

**Affiliations:** Orthodontic Section, Department of Preventive Dentistry, Faculty of Dentistry, Prince of Songkla University, Hat Yai 90110, Songkhla, Thailand; siangloy.th@gmail.com

**Keywords:** bimaxillary protrusion, incisal inclination, incisal position, hard tissue, soft tissue, lateral cephalometric

## Abstract

The objective of this study was to investigate the upper incisors (U1), lower incisors (L1), and soft tissue profiles of bimaxillary protrusion (BM) adult patients among skeletal Class I (BM1), II (BM2) and III (BM3). Understanding these characteristics would be useful for incisor and lip diagnostics in different skeletal classifications. Fourteen linear and twelve angular variables of the incisors and lips were evaluated in 214 lateral cephalograms (BM1 = 91, BM2 = 84, BM3 = 39). ANOVA and Bonferroni tests compared the measurements. BM1 and BM3 exhibited a greater U1 position and U1 inclination than the norms, while BM2 presented only a greater U1 position than the norms but normal U1 inclination. BM1 and BM3 had a significantly greater U1 position than BM2. BM1 and BM2 demonstrated a greater L1 position and L1 inclination than the norms, whereas BM3 displayed only a greater L1 position than the norms but normal L1 inclination. BM2 had the most anterior L1 position, whereas BM3 had the least anterior position. Only BM2 had a longer anterior dental height (ADH) than the norms, while BM1 and BM3 had a normal ADH and the significantly shortest ADH, respectively. Only BM1 had a normal upper incisor display at rest (U1R), while BM2 and BM3 displayed an increased and decreased U1R, respectively, with significant differences among the three groups. The most significantly protruded upper and lower lips were presented in BM2, but these were exhibited the least in BM3. The most significant acute nasolabial angle (NLA) was found in BM3, whereas BM2 presented the least acute NLA. A normal lip–chin–throat angle (LCTA) was observed in BM1 and BM3, while only BM2 had a greater LCTA than the norms. The most significant obtuse LCTA was found in BM2, while BM3 had the least obtuse LCTA. Therefore, both U1 and L1 in all groups presented protrusion and proclination, except for U1 in BM2, while L1 in BM3 exhibited normal inclination. The ADH and U1R were increased in BM2 but decreased in BM3. The most acute NLA was found in BM3, whereas the least acute was found in BM2. The most obtuse LCTA was in BM2, while the least was in BM3.

## 1. Introduction

Bimaxillary protrusion (BM) is one of the unaesthetic appearances exhibited by patients who seek orthodontic treatment. This appearance is not only caused by the protruded upper and lower lips, but also the protruding upper and lower incisors. The underlying protruded anterior teeth are considered the cause of lip protrusion since they cover the teeth. BM can be found in all skeletal classifications, including Class I, II, and III, which are designated as BM1, BM2, and BM3. Each has their own incisor and lip characteristics. Various ethnicities and races have different facial features. Research conducted in adult subjects would be more beneficial than that conducted in adolescents since growth is not an issue [1].

In BM, not only are the incisors in a too anterior position, known as protrusion, but they also have projected inclination, known as proclination. Most previous bimaxillary protrusion studies [2,3,4,5,6,7,8] have enrolled subjects with skeletal Class I malocclusion. Only one study has been conducted in each of skeletal Class II [9] and III [10]. In previous research on BM1, four of the studies [3,4,5,8] reported upper incisor (U1) and lower incisor (L1) bimaxillary protrusion, as the incisors should be, and only three of them studied U1 and L1 inclination [3,4,5]. However, two studies [2,6] found only L1 protrusion and proclination, whereas U1 had a normal position and inclination. Some studies lacked the anteroposterior skeletal parameters to identify BM classifications [3,7]. Few studies have presented the norms and the amount of protrusion compared to the norms [2,4]. Aldree et al. [4] used the Saudi norms for U1 position (U1-NA (mm)) and U1 inclination (U1-NA(°)), which were approximately 4.44 mm and 21.82°, and for L1 position (L1-NB (mm)) and L1 inclination (L1-NB (°)), which were 5.34 mm and 26.04°; meanwhile, Lamberton et al. [2] used Steiner’s norms for U1-NA, which were approximately 4 mm and 22°, and for L1-NB, which were 4 mm and 25°. When comparing the Saudi and Steiner norms (mm, degree), the U1-NA was comparable, but L1-NB in the Saudi control showed more proclination and protrusion than Steiner’s norm. Moreover, the greatest protrusion of U1-NA (10.2 mm) and L1-NB (11.5 mm) was presented in only one study, but without soft tissue investigations [5]. Unfortunately, for the BM2 group, only one study [9] that investigated skeletal, dental, and soft tissue parameters has been conducted. Nevertheless, their subjects had no U1 protrusion and the protrusion was exhibited only in L1. Similarly, only one BM3 study [10] was found. This investigated only skeletal and dental parameters without soft tissue measurements. It found both U1 and L1 protrusion but only U1 proclination. From those previous studies, there is controversy and a lack of knowledge regarding the incisor position and inclination in BM1, BM2, and BM3. Therefore, conducting investigations in these groups of BM is interesting since no comparison has been conducted among BM1, BM2, and BM3. Furthermore, vertical dental parameters such as the anterior dental height (ADH) and upper incisor display at rest (U1R) are very important for investigation in aesthetic assessments.

The upper and lower lip characteristics in BM are usually reported by comparing their position and angulation to the nose and the chin. The positions of the upper and lower lips are frequently evaluated by referring to the E plane, which is a line between the nose and chin. The angulation of the lips is evaluated using the nasolabial angle (NLA) for the upper lip and the lip–chin–throat angle (LCTA) is used for the lower lip. In general, the upper and lower lip of BM subjects are in front of the E plane. Therefore, the NLA is regularly acute while the LCTA is usually obtuse compared to the norms. In BM1 subjects, the upper and lower lips are also beyond the E plane. Out of eight NLA studies in BM1 subjects, three studies [6,8,11] showed acute NLA (77.6°–88.88°) while five studies [4,6,7,12,13] showed obtuse NLA (93.9°–110.34°). In BM2, only one study [9] was conducted with obtuse NLA (101.4°), while no study has reported NLA results for BM3. The LCTA has not been reported in any BM subjects. Other related soft tissue parameters, e.g., the upper lip length (ULL), lower lip length (LLL), upper facial height (UFH), lower facial height (LFH), and interlabial gap (ILG), are also investigated in this current research, and these are all useful for an aesthetic evaluation.

Since a gap in the knowledge exists, this study investigated BM in three different skeletal classifications in adult subjects. The objectives were (1) to compare the upper and lower incisor positions and inclinations, and (2) to compare the upper and lower lip positions and angulations. Understanding these characteristics can be useful for diagnostic purposes in BM patients with different skeletal classifications.

## 2. Materials and Methods

### 2.1. Subjects

This was a retrospective study conducted in 214 Thai adult patients (101 males and 113 females). Ethical approval was obtained from the Human Ethics Committee of the Faculty of Dentistry, Prince of Songkla University (protocol number EC6411-069). Patients who presented with upper and lower lip protrusion who sought orthodontic treatment at the orthodontic clinic, Faculty of Dentistry, Prince of Songkla University, Songkhla, Thailand, between 2012 and 2021 were included in the study. The inclusion criteria were as follows: (1) adult patients aged 18–35 years old; (2) protrusive upper and lower lips with an initial lip protrusion greater than the norms (3.2 ± 1.96 mm for upper lip, and 2.80 ± 2.83 mm for lower lip) according to Ricketts’ E-plane for lip position—the plane formed by joining the tip of the nose and the soft tissue pogonion [14]; (3) a normal overjet (OJ) of 2–4 mm; (4) well-aligned teeth or minimal crowding; and (5) the presence of a good-quality pretreatment lateral cephalogram. The exclusion criteria were as follows: (1) previous orthodontic treatment; (2) history of trauma or surgery involving facial structures; (3) craniofacial deformities or syndromes, e.g., cleft lip and palate; (4) abnormalities in the anterior teeth, e.g., missing tooth, impacted tooth, prostheses or large restorations extending to incisal edges; and (5) periodontal or gingival diseases.

### 2.2. Cephalometric Analysis

**Figure 1:**
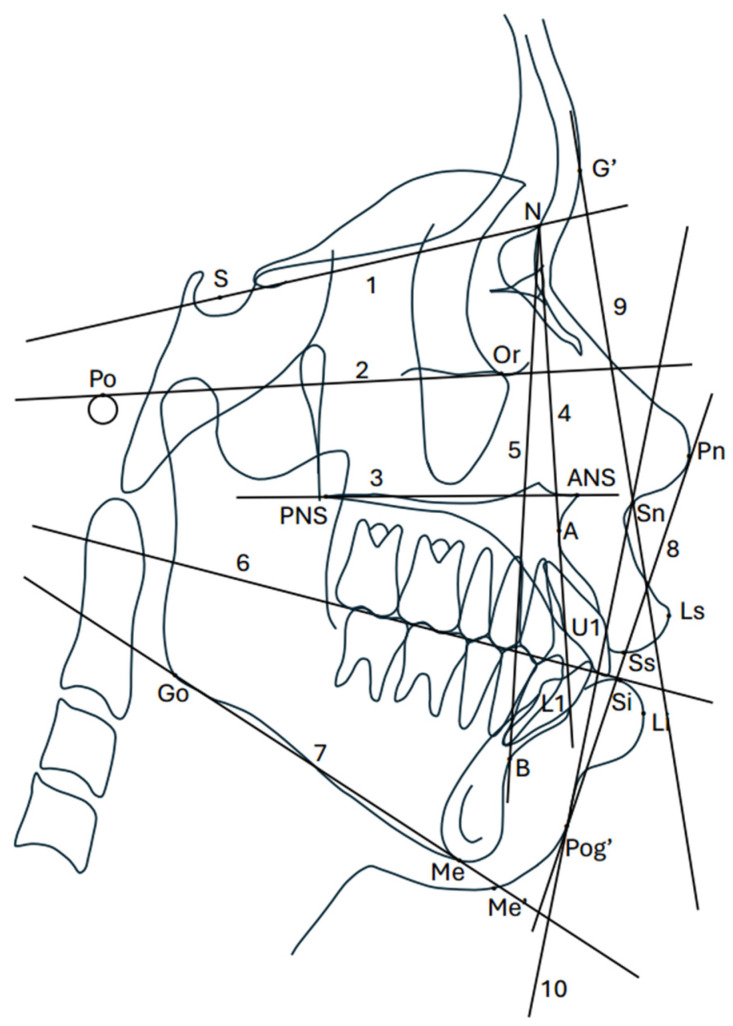
Cephalometric landmarks and reference planes in this study. S, sella; N, nasion; Po, porion; Or, orbital; ANS, anterior nasal spine; PNS, posterior nasal spine; A, A point; B, B point; Go, gonion; Me, menton; U1, maxillary incisor; L1, mandibular incisor; Ls, labrale superius; Li, labrale inferius; Sn, subnasale; Pn, pronasale; G’, soft tissue glabella; Pog’, soft tissue pogonion; Me’, soft tissue menton; Ss, stomion superius; Si, stomion inferius; 1, SN; 2, FHP; 3, PP; 4, NA; 5, NB; 6, FOP; 7, MP; 8, EP; 9, UFP; and 10, LFP.

Pretreatment lateral cephalograms were taken from the same X-ray machine, namely Orthopantomograph^®^ OP300 (Instrumentarium Dental, Tuusula, Finland), at 90 kV, at 12.5 mA, using a 15 s exposure time, and at a magnification 10.45%. All radiographs were obtained in the natural head position. The subjects stood straight and were asked to place their arms at their sides to establish the “orthoposition”. They were then instructed to close their eyes. After a series of neck-bending exercises, tilting the head upward and downward by decreasing the amplitude until a comfortable position of natural balance was achieved, the subjects reopened their eyes and looked into their reflected eyes on a mirror placed 3 feet away. When the patient was in the natural head position, the patients’ visual axis was parallel to the floor [15]. Teeth were occluded in the maximum intercuspation, and lips were maintained in a relaxed position. Cephalometric tracing was digitized and analyzed using Dolphin Imaging^®^ (version 11.9; Dolphin Imaging, Chatsworth, CA, USA). The measurements from the cephalograms were converted to actual distances. A scale ruler on the cephalogram was used to perform the mathematical conversion using ImageJ software (version 1.53a NIH, Bethesda, MD, USA). The standard cephalometric landmarks and reference planes are shown in Figure 1. Table 1 includes 26 measurements (14 linear and 12 angular) of the skeletal, dental, and soft tissue that were obtained from the lateral cephalograms. Linear measurements were reported in millimeters (mm) and angular measurements in degrees (°).

### 2.3. Sample Size Calculation

Since similar studies do not exist, a pilot study was conducted in 20 subjects randomly selected from each of the three BM groups. The patient selection was simple random sampling using random numbers. The most significant difference found in the pilot study was the L1-NB inclination of 0.017. The means and standard deviations from this parameter were used to calculate the sample size. The sample size was calculated using G*Power program version 3.1 (Franz Faul; Christian-Albrechts-Universitat, Kiel, Germany), based on a significance level of alpha = 0.05 and power = 80%. The effect size of the pilot study was 0.216 for sample size calculation. Therefore, the total sample size of this study was 210. At least 70 subjects were required per group. However, the number of subjects in BM3 was limited to 39. The final numbers of subjects in BM1 and BM2 were 91 and 84, respectively, for a better representation of the population.

**Table 1:** Definitions of cephalometric landmarks, reference planes, and measurements.

Measurements	Definitions
Cephalometric landmarks	
Sella; S	The midpoint of the Sella turcica (pituitary fossa).
Nasion; N	The most anterior point on the frontonasal suture.
Orbitale; Or	The most anterior, inferior point on the infraorbital rim.
Porion; Po	The upper midpoint on the external auditory meatus.
Anterior nasal spine; ANS	The tip of the anterior nasal spine.
Posterior nasal spine; PNS	The tip of the posterior nasal spine.
A point; A	The point of the deepest concavity anterior curvature of the maxillary alveolus.
B point; B	The point of the deepest concavity anterior on the mandibular symphysis.
Gonion; Go	The most posterior and inferior point on the mandibular angle.
Menton; Me	The most inferior point on the mandibular symphysis.
Upper incisors; U1	The most anteriorly positioned maxillary incisor.
Lower incisors; L1	The most anteriorly positioned mandibular incisor.
Labrale superius; Ls	The most anterior point on the convexity of the upper lip (UL).
Labrale inferius; Li	The most anterior point on the convexity of the lower lip (LL).
Subnasale; Sn	The point in the midsagittal plane where the base of the columella of the nose meets the upper lip.
Pronasale; Pn	The most prominent and anterior point of the nose.
Soft tissue glabella; G’	The most anterior point of the soft tissue in the forehead.
Soft tissue pogonion; Pog’	The most anterior point of the chin.
Soft tissue menton; Me’	The lowest point on the contour of the soft tissue chin.
Stomion superius; Ss	The lowermost point of the upper lip vermillion border.
Stomion inferius; Si	The uppermost point of the lower lip vermillion border.
Reference planes	
SN plane; SN	The plane demonstrated by a line through the sella (S) and nasion (N).
Frankfort horizontal plane; FHP	The plane demonstrated by a line through the porion (Po) and orbitale (Or).
Palatal plane; PP	The plane demonstrated by a line through the anterior nasal spine (ANS) and posterior nasal spine (PNS).
NA plane; NA	The plane demonstrated by a line through the nasion (N) and A point.
NB plane; NB	The plane demonstrated by a line through the nasion (N) and B point.
Functional occlusal plane; FOP	The plane demonstrated by a line through the occlusion between premolars and molars.
Mandibular plane; MP	The plane demonstrated by a line through the gonion (Go) and menton (Me).
Ricketts’ E plane; EP	The plane formed by joining the tip of nose and soft tissue pogonion (Pog’) [14].
Upper facial plane; UFP	The plane demonstrated by a line through the soft tissue glabella (G’) and subnasale (Sn).
Lower facial plane; LFP	The plane demonstrated by a line through the subnasale (Sn) and soft tissue pogonion (Pog’).
Skeletal measurements	
SNA	The angle formed by a line through the SN plane and A point, which represents the relative anteroposterior position of the maxilla to the cranial base.
SNB	The angle formed by a line through the SN plane and B point, which represents the relative anteroposterior position of the mandible to the cranial base.
ANB	The angle formed by lines connecting the A point, nasion, and B point. It represents the relative anteroposterior position of the maxilla to the mandible, which determines the skeletal classification.
Mandibular plane angle; MPA	The angle between the Frankfort horizontal plane (FHP) and the mandibular plane (MP), indicating the vertical mandibular growth pattern.
Dental measurements	
U1-NA position; U1-NA (mm)	The perpendicular distance from the incisal edge of the most anteriorly positioned maxillary incisor (U1) to the NA plane, representing the position of the upper incisors.
U1-NA inclination; U1-NA (°)	The angle formed by the axis of the most anteriorly positioned maxillary incisors (U1) and the NA plane, representing the inclination of the upper incisors.
L1-NB position; L1-NB (mm)	The perpendicular distance from the incisal edge of the most anteriorly positioned mandibular incisor (L1) to the NB plane, representing the position of the lower incisors.
L1-NB inclination; L1-NB (°)	The angle formed by the axis of the most anteriorly positioned mandibular incisors (L1) and the NB plane, representing the inclination of the lower incisors.
Interincisal angle; U1-L1	The angle between the axis of the most anteriorly positioned maxillary (U1) and the mandibular incisors (L1).
U1-PP degree; U1-PP	The angle between the axis of the most anteriorly positioned maxillary incisor (U1) and the palatal plane (PP).
L1-MP degree; L1-MP	The angle between the axis of the most anteriorly positioned mandibular incisor (L1) and the mandibular plane (MP).
Anterior dental height; ADH	The distance from the ANS to the incisal tip of the most anteriorly positioned maxillary incisor (U1) perpendicular to the SN plane (SN).
Upper incisor display at rest; U1R	The distance from the stomion superius (Ss) to the incisal tip of the most anteriorly positioned maxillary incisor (U1), representing the maxillary incisor display at rest position [16].
Overjet; OJ	The horizontal overlap of the incisors.
Overbite; OB	The vertical overlap of the incisors.
Curve of Spee; COS	The perpendicular distance from the deepest cusp tip of the bicuspid to functional occlusal plane (FOP).
Soft tissue measurements	
Upper lip to E plane; UL-EP	The distance from the most anteriorly positioned upper lip to Rickett’s E-line, representing the protrusion of the upper lip.
Lower lip to E plane; LL-EP	The distance from the most anteriorly positioned lower lip to Rickett’s E-line, representing the protrusion of the lower lip.
Nasolabial angle; NLA	The angle between the pronasale (Pn), subnasale (Sn), and upper vermilion of the lip.
Lip chin throat angle; LCTA	The angle formed by a line between the lower border of the chin and a line connecting the lower lip and soft tissue pogonion (Pog’).
Facial contour angle; FCA	The angle formed by the intersection between the upper facial plane (UFP) and the lower facial plane (LFP).
Upper lip length; ULL	The distance from the subnasale (Sn) to stomion superius (Ss).
Lower lip length; LLL	The distance from the stomion inferius (Si) to soft tissue menton (Me’).
Upper facial height; UFH	The distance from the soft tissue glabella (G’) and subnasale (Sn).
Lower facial height; LFH	The distance from the subnasale (Sn) to soft tissue menton (Me’).
Interlabial gap; ILG	The distance from the stomion superius (Ss) to stomion inferius (Si) [17]

All lateral cephalograms of subjects were digitized, analyzed, and classified into three groups of BM based on skeletal patterns. Each group was comprehensively determined from the ANB, representing the anteroposterior relationship of the maxilla and mandible. Subjects who had an ANB in normal values (3.20 ± 1.99°) [18] were categorized as BM1. Subjects who had an ANB greater than the normal values were categorized as BM2. Subjects who had an ANB less than the normal values were categorized as BM3. The characteristics of the subjects are shown in Table 2.

### 2.4. Statistical Analysis

All measurements were performed by one examiner blinded to the other film of the same subject. Thirty randomly selected subjects were remeasured after two weeks to determine measurement error and reliability. Comparisons between the first and second measurements that used the independent *t*-test and intraclass correlation coefficient (ICC) illustrated no significant differences between the first and second measurements from the cephalograms. To determine reliability, ICC values less than 0.5 were indicative of poor reliability, values between 0.5 and 0.75 indicated moderate reliability, values between 0.75 and 0.9 indicated good reliability, and values greater than 0.9 indicated excellent reliability [19]. No systematic error was observed for any variables. Furthermore, random errors were estimated by Dahlberg’s formula [20], which exhibited measurement errors of 0.11 mm for the linear measurement and 0.20° for the angular measurement in the cephalometric measurements. These random errors were considered acceptable.

SPSS version 26.0 (IBM Corp., Armonk, NY, USA) was used for the statistical analysis of the data. The Kolmogorov–Smirnov statistics test demonstrated normal distributions in BM1 and BM2. The Shapiro–Wilk test exhibited normal distributions in BM3. Descriptive statistics (mean and the standard deviation) were determined and used to assess the skeletal, dental, and soft tissue variables compared to normal Thai values [18,21,22]. One-way analysis of variance (ANOVA) was performed for a statistical comparison of the skeletal, dental, and soft tissue measurements among the three BM groups. The Bonferroni test [23] was used to pairwise compare between the groups. A *p*-value less than 0.05 was considered statistically significant.

## 3. Results

The intra-rater reliability was excellent. The correlation coefficients for the two examiners were 0.94 for T.S. and 0.96 for C.C. The inter-rater reliability was also excellent. The correlation coefficient was 0.92.

There were no significant differences in the average ages of the three groups. More females than males were in all groups, with comparable proportions of all three groups. The maxillae in all groups were within normal limits. However, the most significant prognathism was observed in BM2, whereas BM3 had the least prognathism. The mandibles in all groups were also within normal limits. Nonetheless, the most significant prognathism was observed in BM3, whereas BM2 had the least prognathism. The MPA of BM1 and BM3 indicated normodivergent facial patterns, but only BM2 showed a hyperdivergent facial pattern. The significantly greatest MPA was demonstrated in BM2, whereas the least MPA was presented in BM3 (Table 2).

Table 3 presents the dental measurements. BM1 and BM3 exhibited a greater U1-NA (mm) and U1-NA (°) than the norms, whereas BM2 presented only a greater U1-NA (mm) than the norms but a normal U1-NA (°). Although all BM groups had protrusion, BM1 and BM3 had a significantly greater U1-NA (mm) than BM2. Meanwhile, all three groups had significant differences in U1-NA (°). BM3 had the most projected inclination, whereas BM2 had the least proclination. U1-PP showed similar characteristics to U1-NA (°) for all groups. BM1 and BM2 exhibited a greater L1-NB (mm) and L1-NB (°) than the norms, whereas BM3 presented only a greater L1-NB (mm) than the norms but normal L1-NB (°). All groups had significant differences in L1-NB (mm). BM2 had the most anterior position, whereas BM3 had the least protrusion. L1-NB (°) and L1-MP were also significantly different. BM2 had the most projected inclination, whereas BM3 had the least proclination. Moreover, for the interincisal angle (U1-L1), the most significant obtuse angle was found in BM3, whereas the least was observed in BM1.

When we consider U1 vertical measurements, only BM2 had a longer ADH than the norms, whereas BM1 and BM3 had normal ADH values. Among the three groups, BM2 presented the significantly longest ADH, while the significantly shortest ADH was observed in BM3. Only BM1 had a normal U1R, while BM2 had a U1R that was too long and BM3 had a U1R that was too short. BM2 showed the significantly longest U1R, whereas BM3 presented the significantly shortest U1R.

The OJ in all three groups was within normal limits. However, only BM2 demonstrated a significantly greater OJ than the other two groups. Only BM1 and BM3 had a normal OB, while BM2 demonstrated a greater OB. Among the three groups, the significantly greatest OB was found in BM2 and the smallest OB was found in BM3. The COS in all three groups was also within normal limits. Among the three groups, the significantly greatest COS was found in BM2, with the lowest COS in BM3.

Both the upper and lower lips indicated protrusion compared to the norms. The most significantly protruded upper lip and lower lip were presented in BM2, but the lowest lip protrusion was found in BM3. The NLA measurements were normal in all groups but more acute compared to the mean average. The most significantly acute NLA was found in BM3, whereas the least acute was presented in BM2. The LCTA was normal in BM1 and BM3, but BM2 had a greater LCTA than the norms. The most significantly obtuse LCTA was in BM2, with the least significantly obtuse LCTA in BM3. The greatest FCA was found in BM2 followed by BM1, with the smallest measurement found in BM3. The ULL measurements in all groups were normal. Among the three groups, the significantly longest ULL was demonstrated in BM2, whereas BM1 and BM3 were comparable. The LLL in all groups was normal. Among the three groups, the significantly shortest LLL was found in BM3, whereas BM1 and BM2 were comparable. The UFH in BM1 and BM3 was normal. Only the UFH in BM2 was longer than the norms. Among the three groups, the significantly longest UFH was in BM2, whereas that in BM3 was significantly the shortest. The LFH measurements in all groups were normal. Among the three groups, the significantly longest and shortest LFH were in BM2 and BM3, respectively. The interlabial gaps (ILG) were wider than the norms in all groups. Among the three groups, the significantly widest and narrowest interlabial gaps were in BM2 and BM3, respectively (Table 4).

## 4. Discussion

The severity of the U1 position and inclination in BM1 (U1-NA 10.16 mm, 32.13°) in this study was greater than most previous studies, which had ranges of 4.57–7.62 mm and 23.80–31.59° [2,4,6,11]. Only the study by Sivakumar et al. [5] was comparable to this study, with U1-NA values of 10.20 mm and 35.10°. In the BM2 group of this study, the U1 position and inclination (U1-NA 9.50 mm, 26.06°) were also greater than those reported in a study by Lee et al. (U1-NA 5.70 mm, 22.80°) [9]. In the BM3 group of this study, the U1 position and inclination (U1-NA 10.72 mm, 36.33°) were greater than in a previous study [10]. It can be implied that the severities of protrusion and proclination in the samples of this study were beyond the results of previous BM research for all three types of skeletal classifications. The U1 characteristics in all groups indicated protrusion, but proclination presented only in BM1 and BM3. The U1 protrusion and proclination found in BM3 were also discovered in a study by Osman and Sethusa [10]. In the BM3 group, the U1 had the most projected inclination or the most proclination among the three groups. Unfortunately, a previous study [10] that showed the same characteristics did not give any possible explanations. Since the maxilla in BM3 was in the most posterior position with the most anterior position of the mandible, the U1 needed to compensate for this intermaxillary deficiency by flaring labially [24,25]. The BM2 group showed normal inclination but not proclination, as in the other two groups. This normal inclination was also exhibited in a prior study in skeletal Class II subjects [9]. However, the U1 results in the Lee et al. study [9] showed a normal position but not protrusion, as in this study. The findings in this study implied that, even though the U1 exhibited more protrusion, the inclination was still normal. This reduced proclination of the U1 indicated compensation to the mandible, which was in the most posterior position [24]. In conclusion, the U1 in both BM1 and BM3 was similar regarding protrusion and proclination, but more proclination was detected in BM3. The BM2 group was unique, with a normal inclination of U1.

The severity of the L1 position and inclination in BM1 (L1-NB 11.85 mm, 38.32°) in this study was greater than most previous studies [2,4,6,11], which had ranges of 6.50–8.20 mm and 27.80–35.10°. Similar to the U1 in BM1, the findings of Sivakumar et al. [5] were consistent with this study, with L1-NB measurements of 11.50 mm and 39.90°. The L1 in all groups indicated protrusion, but proclination was presented only in BM1 and BM2. The L1 in BM3 showed normal inclination without proclination, as in the other two groups. The L1 protrusion with normal inclination in BM3 was also reported in a previous study [10]. This L1 normal inclination that presented particularly in BM3 also indicated dental compensation for skeletal discrepancy [24]. On the contrary, the L1 in BM2 had the most projected inclination or the most proclination among the three groups. Lee et al. found the same findings in L1 protrusion and proclination [9]. Since the maxilla in BM2 was in the most anterior position and the mandible was in the most posterior position, the L1 needed to compensate for this intermaxillary deficiency by flaring labially [24]. In summary, the L1 in both BM1 and BM2 was similar since they both showed protrusion and proclination, but more proclination was detected in BM2. However, BM3 was unalike, due to the L1 in normal inclination. When combining both the U1 and L1 characteristics in all three BMs, both the U1 and L1 exhibited protrusions defined as bimaxillary protrusions. Nevertheless, the inclination tended to be proclination, with specific variations in the U1 in BM2 and the L1 in BM3, which had normal inclination. 

The vertical measurements of the U1 in the BM2 group were longer than the norms, since there could be vertical dental compensation toward the L1 located in the retruded mandible [26,27]. The significantly shortest ADH found in BM3 could have resulted from the obvious proclination of the U1, causing the ADH to become shortened. The long ADH in BM2 and the short ADH in BM3 coincided with the greater exposure of the U1R in BM2 and the lower exposure of the U1R in BM3. On the other hand, the study by Lee et al. indicated a normal UIR in BM2 [9]. The reason the U1R in this study was higher than that reported by Lee et al. [9] was that the samples in this study had a greater SNA and ANB. Greater jaw discrepancies could cause the more vertical compensation of U1, as shown by the greater U1R [26]. Unfortunately, no previous research has focused on the U1R in BM3; only case reports have been published. The first study showed no U1R from the tracing, with a 46-degree U1-NA [28]. The other study showed an approximately 2 mm U1R from the tracing, with a 32-degree U1-NA [29]. This study had a 0.53 mm U1R, which was greater than that reported by Verma [23] but less than Bellamine and Ousehal [24]. A possible explanation was U1 inclination, which in this study had less proclination compared to Verma [23] but more proclination compared to Bellamine and Ousehal [24]. Therefore, this could imply that greater U1 proclination leads to a smaller U1R. Again, the ADH was associated with the U1R [30]. In conclusion, the ADH and U1R in BM2 were long in contrast to BM3, where the ADH and U1R were short. 

Among the three groups, OB was the greatest in BM2 and was the lowest in BM3, which was similar to the COS. Nayar et al. [31] reported a correlation between the COS and OB among Class I, Class II, and Class III malocclusion groups. Furthermore, they revealed that the COS was the deepest in Class II subjects and flatter in Class III subjects. In addition, this current study found that the greatest OB in BM2 coincided with its greatest ADH and COS. Likewise, the lowest OB in BM3 coincided with its shortest ADH and COS [32]. 

When we consider soft tissue anteroposterior characteristics, the most significantly protruded upper lip and lower lip were presented in BM2, but the least were exhibited in BM3. The amount of lip protrusion can be caused by either the lips themselves or the different locations of the landmarks forming the reference planes. In this case, it was the mandible (SNB) in the BM2 group that had a more posterior position in contrast to BM3, which had the most anterior mandibular position. Therefore, the reference line for lip position measurements would be more posterior in the BM2 group but more anterior in BM3, which would cause more lip protrusion in BM2 but less in BM3. The most significantly acute NLA was found in BM3, whereas the least was presented in BM2. Only one previous study [9] presented an NLA of 101.4° in BM2, which was greater than this study (86.14°). The NLA is related to the upper incisor, which is located underneath the upper lip [33]. We found that the U1-NA position and degrees in a former study [9] were 5.7 mm and 22.8°, respectively, which was less than this study that demonstrated 9.50 mm and 26.06°, respectively. Conversely, only one research paper [10] studied BM3, but it did not perform a soft tissue investigation; therefore, a comparison was not possible. From a BM3 case report [28], the patient presented an acute NLA of 72°, which was less than this study (82.65°). The explanation is similar to the BM2 aforementioned reason, where the U1 of the case report had more proclination (U1-NA 46°) than this study (U1-NA 36.33°). Additionally, the most significantly obtuse LCTA was in BM2 and the least significantly obtuse LCTA was in BM3. In contrast, the LCTA was not investigated in either BM2 or BM3 for comparison. This angle was also affected by the hard tissues underneath the soft tissue, which were the L1 and the mandible. Therefore, the most anterior position and proclination of L1 with the most posterior position of the mandible in BM2 could be the cause that resulted in the most obtuse LCTA. 

The significantly longest ULL was shown in BM2, whereas BM1 and BM3 were comparable. The longest ULL in BM2 could be related to the smallest U1 protrusion and proclination; therefore, the UL was not pushed up by the U1 to become shortened. Skeletal hyperdivergent BM2 could be another possible reason for a longer ULL, since a hyperdivergent growth pattern indicated more vertical growth [34]. The significantly shortest LLL that was shown in BM3 has never previously been reported; therefore, an explanation and comparison was not possible. Furthermore, the significantly longest UFH was found in BM2, whereas the significantly shortest UFH was observed in BM3. These can be explained by skeletal divergence. The greatest MPA of BM2 indicated more vertical growth, whereas the smallest MPA of BM3 indicated less vertical growth [35]. Similar findings indicated that the significantly longest and significantly shortest LFH were observed in BM2 and BM3, respectively. The explanation for this would be the same as previously mentioned for UFH. Moreover, the ILG was found to be significantly widest and narrowest in BM2 and BM3, respectively. Consequently, the skeletal vertical pattern would be associated with this ILG, since a hyperdivergent pattern can produce more posterior mandibular rotation [36], which causes the lips to be more apart in BM2 but less apart in BM3, in which the divergence (MPA) was less than in the other groups. 

The clinical implications from this study include both a diagnosis and treatment plan in orthodontics. The incisor characteristics among the BM groups were unique. Clinicians should be aware of these characteristics when making a diagnosis in orthodontics. A typical feature of BM is U1 and L1 protrusion and proclination, but this is not the case for the U1 in BM2 and L1 in BM3, which are protruded but with normal inclination. Therefore, clinicians must be aware when diagnosing the U1 in BM2 and L1 in BM3. In general, the treatment plan for BM with U1 and L1 protrusion and proclination should include retraction with tipping movement in both the U1 and L1. However, in BM2, the U1 should be retracted with bodily movement and the L1 should be retracted with tipping movement. In BM3, the U1 should be retracted with tipping movement while the L1 should be retracted with bodily movement. In addition, for the U1R, an excessive U1R found in BM2 should be intruded, which would be the opposite in the case of a short U1R in BM3, which should be extruded.

The limitations of this study need to be acknowledged. First, the research was conducted in patients aged 18–35 years, since growth ceases at 18 years [37] and late soft tissue change occurs over 35 years of age [38]. Therefore, the findings of the study cannot be applied to patients either younger than 18 or older than 35 years. Second, regarding the subject’s vertical patterns, BM1 and BM3 are normodivergent facial patterns, and BM2 is a hyperdivergent facial pattern. Thus, vertical patterns other than the normodivergent pattern in BM1 and BM3 and the hyperdivergent pattern in BM2 cannot be considered. Third, all subjects in this study exhibited orthognathic maxilla and mandible; therefore, the results of this study cannot be applied to prognathic patients. Fourth, the results of the study cannot be applied to patients with an OJ greater or less than 2–4 mm. Finally, the results cannot be generalized to patients with moderate to severe crowding and spacing.

Further studies should evaluate changes in the incisor position, inclination, and soft tissue profile after a reduction in incisor protrusion in specific BM types with different anteroposterior and vertical maxilla–mandibular relationships. Other future studies should be conducted in both growing and older BM patients, with comparisons between males and females.

## 5. Conclusions

The U1 in both the BM1 and BM3 groups showed protrusion and proclination. More proclination was detected in the BM3 group. Meanwhile, the U1 in BM2 revealed protrusion but normal inclination. The L1 in both BM1 and BM2 demonstrated protrusion and proclination. More proclination was detected in BM2. Meanwhile, the L1 in the BM3 group revealed protrusion but normal inclination. The vertical position of the U1 revealed that the ADH and U1R of BM2 were long. On the contrary, the BM3 group showed a short ADH and U1R. The most significantly protruded UL and LL were presented in BM2, but the least were exhibited in BM3. The most significant acute NLA was found in the BM3 group, whereas the least was presented in the BM2 group. The most significant obtuse LCTA was shown in the BM2 group, with the least significantly obtuse LCTA in the BM3 group.

## Figures and Tables

**Table 2:** Demographic data of the subjects (*N* = 214).

Characteristics	Norms	BM1 (*n* = 91)	BM2 (*n* = 84)	BM3 (*n* = 39)	*p*-Value
Age, y	N/A	25.44 (0.44)	24.97 (0.30)	25.37 (0.34)	0.639
Gender	N/A				N/A
Female/Male		47/44	45/39	21/18	
Proportion		1:1.07	1:1.15	1:1.17	
SNA, °	85.02 (3.73)	85.34 (0.15)	87.19 (0.13)	83.57 (0.33)	<0.001 * *^a,b,c^*
SNB, °	81.78 (3.28)	81.96 (0.24)	79.94 (0.19)	84.92 (0.34)	<0.001 * *^a,b,c^*
ANB, °	3.20 (1.99)	3.39 (0.25)	7.05 (0.22)	−1.75 (0.35)	<0.001 * *^a,b,c^*
MPA, °	22.74 (5.37)	26.38 (0.23)	31.75 (0.26)	20.97 (0.23)	<0.001 * *^a,b,c^*

Values are presented as mean ± standard deviation; Refer to Table 1 for the definition of each measurement. Differences between groups were tested by ANOVA and pairwise comparisons were performed using the Bonferroni test.; *^a^*, BM1 compared to BM2; *^b^*, BM1 compared to BM3; *^c^*, BM2 compared to BM3.; * *p* < 0.05.

**Table 3:** Comparison of dental measurements of BM in different skeletal types.

Measurements	Norms	BM1 (*n* = 91)	BM2 (*n* = 84)	BM3 (*n* = 39)	*p*-Value
U1-NA (mm), mm	3.39 (1.99)	10.16 (0.14)	9.50 (0.12)	10.72 (0.13)	0.031 * *^a,c^*
U1-NA (°), °	21.58 (4.99)	32.13 (0.20)	26.06 (0.16)	36.33 (0.41)	<0.001 * *^a,b,c^*
L1-NB (mm), mm	6.42 (2.13)	11.85 (0.14)	12.78 (0.20)	10.78 (0.13)	0.027 * *^a,b,c^*
L1-NB (°), °	30.22 (5.57)	38.32 (0.44)	41.45 (0.42)	35.14 (0.18)	<0.001 * *^a,b,c^*
U1-L1, °	124.36 (7.56)	108.32 (3.61)	110.73 (4.52)	112.35 (6.98)	0.035 * *^a,b,c^*
U1-PP, °	119.22 (4.86)	122.25 (5.92)	119.09 (5.24)	125.83 (4.89)	0.022 * *^a,b,c^*
L1-MP, °	99.12 (5.17)	102.86 (5.87)	105.22 (4.94)	100.74 (5.07)	0.019 * *^a,b,c^*
ADH, mm	29.34 (2.54)	31.98 (1.16)	33.47 (1.13)	26.14 (1.12)	<0.001 * *^a,b,c^*
U1R, mm	2.63 (1.15)	2.95 (0.57)	3.97 (1.23)	0.53 (0.11)	0.011 * *^a,b,c^*
OJ, mm	1.98 (0.85)	2.49 (0.71)	2.55 (0.56)	2.24 (0.63)	0.037 * *^a,c^*
OB, mm	1.88 (0.85)	2.67 (0.83)	4.12 (0.64)	2.06 (0.52)	0.039 * *^a,b,c^*
COS, mm	2.02 (0.78)	1.82 (0.11)	1.95 (0.14)	1.71 (0.10)	0.048 * *^a,b,c^*

Values are presented as mean ± standard deviation; Refer to Table 1 for the definition of each measurement. Differences between groups were tested by ANOVA and pairwise comparisons were performed using the Bonferroni test.; *^a^*, BM1 compared to BM2; *^b^*, BM1 compared to BM3; *^c^*, BM2 compared to BM3.; * *p* < 0.05.

**Table 4:** Comparison of soft tissue measurements of BM in different skeletal types.

Measurements	Norms	BM1 (*n* = 91)	BM2 (*n* = 84)	BM3 (*n* = 39)	*p*-Value
UL-EP, mm	−1.23 (1.91)	3.55 (0.87)	4.75 (0.91)	2.65 (0.67)	0.037 * *^a,b,c^*
LL-EP, mm	1.68 (2.03)	6.13 (1.94)	7.42 (1.38)	5.37 (0.52)	0.029 * *^a,b,c^*
NLA, °	90.78 (8.27)	84.03 (5.23)	86.14 (5.79)	82.65 (5.12)	<0.001 * *^a,b,c^*
LCTA, °	115.24 (7.16)	120.32 (5.89)	123.97 (6.01)	118.15 (5.69)	<0.001 * *^a,b,c^*
FCA, °	8.78 (4.50)	11.58 (1.78)	14.25 (1.67)	8.67 (1.82)	0.046 * *^a,b,c^*
ULL, mm	23.30 (2.01)	22.59 (1.66)	24.51 (1.85)	22.05 (1.94)	0.024 * *^a,c^*
LLL, mm	45.88 (2.28)	47.74 (1.72)	47.83 (1.97)	45.98 (1.64)	0.035 * *^b,c^*
UFH, mm	48.42 (2.90)	51.27 (1.61)	52.99 (1.85)	50.13 (1.47)	0.043 * *^a,b,c^*
LFH, mm	69.18 (3.53)	69.39 (2.03)	71.12 (2.37)	67.41 (1.95)	0.018 * *^a,b,c^*
ILG, mm	0.19 (1.00)	3.15 (1.12)	4.03 (1.19)	2.31 (0.98)	0.025 * *^a,b,c^*

Values are presented as mean ± standard deviation; Refer to Table 1 for the definition of each measurement. Differences between groups were tested by ANOVA and pairwise comparisons were performing using the Bonferroni test.; *^a^*, BM1 compared to BM2; *^b^*, BM1 compared to BM3; *^c^*, BM2 compared to BM3.; * *p* < 0.05.

## Data Availability

The data presented in this study are available upon request from the corresponding author.
